# Sensorimotor expertise influences perceptual weight judgments during observation of a sport-specific gesture

**DOI:** 10.3389/fspor.2023.1148812

**Published:** 2023-06-22

**Authors:** Andrea Albergoni, Monica Biggio, Emanuela Faelli, Andrea Pesce, Piero Ruggeri, Laura Avanzino, Marco Bove, Ambra Bisio

**Affiliations:** ^1^ Department of Neuroscience, Rehabilitation, Ophthalmology, Genetics and Maternal Child Health, Università degli Studi di Genova, Genoa, Italy; ^2^ Centro Polifunzionale di Scienze Motorie, Università degli Studi di Genova, Genoa, Italy; ^3^ Section of Human Physiology, Department of Experimental Medicine, Università degli Studi di Genova, Genoa, Italy; ^4^ IRCCS Policlinico San Martino, Genoa, Italy

**Keywords:** motor resonance, action observation, motor expertise, sport expertise, weight estimation

## Abstract

This study aimed to investigate the role of sensorimotor expertise in evaluating relative weight of a lifted object during the observation of a sport-specific gesture, namely the deadlift. Fifty-six participants, assigned to three groups according to their experience in weight lifting, powerlifters, CrossFit® practitioners and naïve participants (controls), performed a perceptual weight judgments task. Participants observed videos showing a powerlifter executing a deadlift at the 80%, 90% and 100% of 1 repetition maximum (1RM) and answered a question about the weight of the lifted object. Participants' response accuracy and variability were evaluated. Findings showed that powerlifters were more accurate than controls. No differences appeared between powerlifter and CrossFit® practitioners, and between CrossFit® practitioners and controls. Response variability was similar in the three groups. These findings suggest that a fine sensorimotor expertise specific for the observed gesture is crucial to detect the weight of the object displayed in the observed movement, since it might allow detecting small changes in the observed movement kinematics, which we speculate are at the basis of the object weight recognition.

## 1. Introduction

Scientific literature argues in favor of a common representation for the perception and the production of human movement. The seminal studies from Johansson put a milestone on the fact that humans are able to recognize biological entities based on few motion information during the observation of a point-light display (1, 2). Furthermore, studies using action observation paradigms showed that the perception of human motion can be improved by prior motor activity (3, 4), and that this phenomenon is shaped by the perceptual motor similarities between self and other stimuli (5, 6). These observations find a neurophysiological basis in the mirror neuron system, a network of frontal and parietal areas activated during both motor and perceptual tasks (7, 8). The activation of this system was postulated to give rise to motor resonance, namely the activation of the observer's motor system during action observation (9). Indeed, motor resonance was shown to be influenced by the observer's motor experience (8), which models the individual motor repertoire, and to be prevented when the observer cannot recognize the biological origin of the observed movement (10, 11).

Another key issue to be considered is the role played by the individual's motor repertoire. For instance, the possibility to map the features of the observed action into the own motor repertoire increases motor resonance, as shown by action observation studies concerning both the temporal features of movements (12, 13) and the ability to anticipate the outcome of an observed action (14–16). Studies comparing motor resonance in athletes and novices during the observation a sport gesture showed that motor resonance is greater when observing “known” than “unknown” movements (17–19). Therefore, the possibility to match the observed movement kinematic into the own motor repertoire seems crucial to make prediction about the observed action.

It was also shown that the kinematics of the actor help the observer to infer the property of the object involved in the action, such as its weight (20–24), although it might be not sufficient, as suggested by Grierson and colleagues who put forward the role of the moved object (25). Furthermore, it was shown that also the display of static pictures of specific phases of action has been proven to yield reliable estimation (26). However, whatever the case, information related to the kinematics of movement, although deduced from static pictures, seems to be crucial in inferring the weight of the object. This effect was explained by subsequent neurophysiological researches showing that the difference in actor's kinematics, when lifting heavy and light objects, modulates the primary motor cortex excitability, and thus motor resonance (27–29).

On the basis of these results, and starting from the notion that sensorimotor expertise modulates motor resonance during action observation, one may hypothesize that individuals who developed specific ability in weight lifting can evaluate the relative weight of the object raised by an observed actor more accurately than non-experts given that the observed movement belongs to their motor repertoire.

Practice of several sports such as powerlifting and CrossFit® includes weightlifting and routinely requires a specific weight training. In powerlifting, athletes are engaged in lifting the maximum possible weight in three specific exercises: the back squat, the bench press and the deadlift (30). In CrossFit®, the training is organized in daily workouts including metabolic exercises, gymnastic movements and weightlifting, thus developing not only strength but also other physical components (31) with a high catabolic impact (32).

The purpose of this study was to investigate whether sensorimotor expertise influences the perceptual weight judgments during the observation of a sport-specific gesture. To this aim, we examined if expert athletes in executing the deadlift (powerlifters and CrossFit® practitioners) manifested higher ability in weight perception judgments than non-experts when observing this specific gesture performed by athletes who lifted a barbell with different weights corresponding to different percentages of their 1 repetition maximum (1RM). Indeed, whilst it is known that motor resonance is differently modulated according to the individual's sensorimotor expertise, the question concerning the role of motor resonance in evaluating the property of an object involved in an observed action deserves to be investigated. Powerlifters were enrolled due to their specific ability in the deadlift (30), while CrossFit® practitioners were recruited as this exercise is a part of their training program (31). We hypothesized that the ability to judge the relative weight of a lifted load was higher in expert subjects than in naïve ones. Furthermore, considering the different level of expertise in the deadlift between powerlifters and CrossFit® practitioners, possible differences could arise between these two categories of expert athletes. We also considered possible differences among the conditions with different weights since, below the 1RM, participants with no experience in weight lifting can have more difficulties in estimating the observed relative weight than experts, whilst at 1RM the effort of the model might help them.

## 2. Materials and methods

### 2.1. Participants

An *a priori* power analysis was conducted using G*Power version 3.1.9.7 (33) to determine the minimum sample required to test the study hypothesis. The effect size was set at 0.25, considered to be medium using Cohen's criteria (34). A *F*-test was applied with a significance criterion of *α* = 0.05 and power = 0.80, the number of groups = 3, number of conditions = 3, the minimum sample size needed with this effect size was *N* = 36 for detecting differences in accuracy.

Fifty-six volunteers participated in the experiment. Based on their sport practice, participants were assigned to three groups: powerlifters (*n* = 18; PL, 5 females and 13 males), CrossFit® practitioners (*n* = 15; CF, 2 females and 13 males) and Controls (*n* = 23, CTRL, 3 females, 20 males). The number of participants in the three groups was motivated by the opportunity we had in the recruitment process. The same reason explained why each sample had largely more males than females, a condition that possibly could influence the results, and for this reason could be a limitation of the study. In the Control group, subjects practiced no activities or activities not related to the weightlifting (Table 1). Furthermore, none of them reported having visual experience with deadlifts. All subjects were fully informed about the study aims and procedures and gave their informed consent. The study was conducted in accordance with the Declaration of Helsinki and the protocol was approved by the Ethics Committee of the University of Genoa (Comitato Etico per la Ricerca di Ateneo, protocol no 2021/42, date of approval 14/04/2021).

**Table 1:** Characteristics of participants.

	Group			Statistical analysis
	Powerlifters	CrossFit® practitioners	Controls	
Number of subjects	18	15	23	-
Age (years)	33 ± 3	28 ± 1	31 ± 2	H(2) = 4.15, *p* = 0.13, *η*^2 ^= 0.04, 95% CI [−0.03 to 0.23]^a^
Sex	Females (5) Males (13)	Females (2) Males (13)	Females (3) Males (20)	
Years of practice	5 ± 1	4 ± 1	-	*U* = 103, *p* = 0.24, *r* = 0.21, CI [0.01–0.51]^b^
Sports practiced	Powerlifting	CrossFit®	Fitness (3) Football (3) Running (2) Basketball (1) Cycling (1) Pilates (1) Rugby (1) Swimming (1) Thai boxe (1) No sport (9)	-
Deadlift 1RM (kg)	220 ± 10	161 ± 10	-	*t*(31) = 3.65, *p* = .001^c^, *g* = 1.24, CI [0.49–1.97]
Level of performance	Agonists (14) Amateur (4)	Agonists (9) Amateur (6)	-	-

Data are mean ± SE, or as number of occurrences. 1RM, 1 repetition maximum.

^a^ Kruskal Wallis Test.

^b^ Mann-Whitney Test between powerlifters and CrossFit® practitioners.

^c^ Unpaired *t*-test between powerlifters and CrossFit® practitioners.

### 2.2. Experimental paradigm

**Figure 1:**
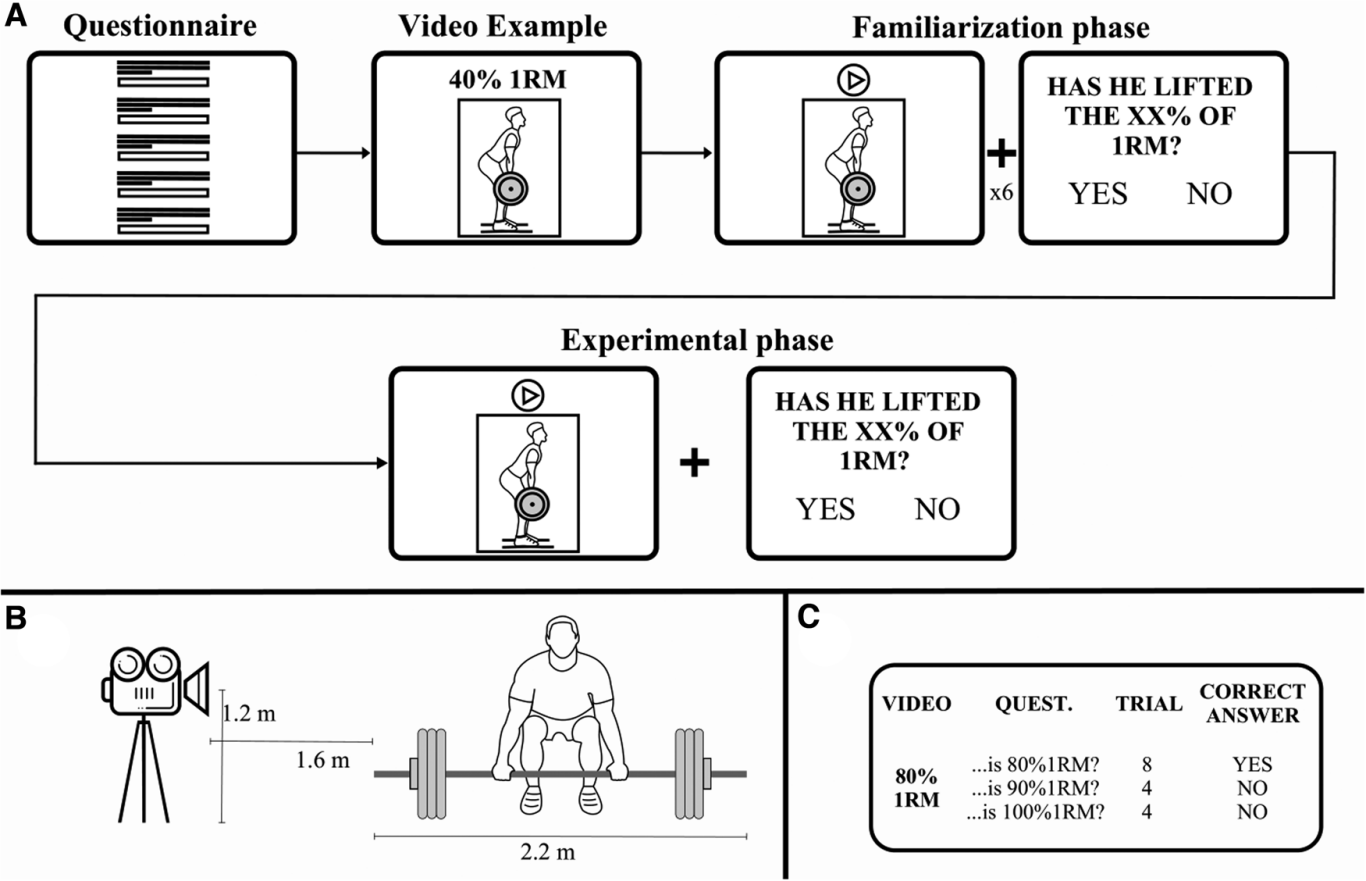
Experimental paradigm. (**A**) Each participant executed the experiment at computer. The participants filled a questionnaire that collected personal and sport-related data. After that, a video example of the deadlift was shown and a familiarization phase, consisting of 6 videos, was performed. Then, participants executed the experimental task. It consisted of 48 videos and a relative question about the magnitude of the weight lifted by the athlete. The videos showed a deadlift performed with three different weights corresponding to 80%, 90% and 100% of athlete's 1RM (16 videos/weight). After each video, the correct answer for each weight were 8 times “Yes” and 8 times “No”. (**B**) Illustration of the set-up during video recording. (**C**) Example of the questions asked to the participants (QUEST.), the number of repetitions for each question (TRIAL) and the correct answers when participants observed the video showing a weight corresponding to 80%1RM.

The experiment was built using jsPsych 6.3.0 library (35). The experimental design included: a questionnaire, a video example, a familiarization phase and the experimental task. The questionnaire collected personal and sport-related data, such as activity performed, years of practice, deadlift 1 repetition maximum (1RM, for PL and CF) (Table 1). After completing the questionnaire, participants observed a video example showing a powerlifter executing a deadlift at 40%1RM. Then, a familiarization phase consisting of observing 6 videos, randomly chosen among those used in the experimental task and followed by relative questions, was performed. Familiarization trials were not included in the analysis. Although this kind of familiarization procedure was already adopted in all the experiments described in Auvray et al. studies on perceptual weight judgments (24), one cannot exclude that it could have influenced the perception of the following movements. For this reason, it could be a limitation. Finally, participants executed the experimental task consisting of watching videos and answering the relative questions about the weight of barbell (Figure 1A).

#### 2.2.1. Visual stimuli

The visual stimuli were videos showing a powerlifter executing a deadlift using the conventional technique, and with three different weights charged on the barbell. In the videos, the athlete acting as a model was a 25-years-old male powerlifter and certified trainer. The weights were determined starting from the maximum weight lifted by the athlete on shoot day, namely his 1RM, corresponding to the 100%1RM condition (210 kg). The other two weights corresponded to the 90%1RM (190 kg) and 80%1RM (167.5 kg) conditions. All videos were recorded on the same day with a recovery period between repetitions and lasted 5.0s (80%1RM), 5.6s (90%1RM) and 6.7s (100%1RM), respectively. During the execution of the deadlift, the model was filmed with a video-camera located in a lateral position so as to record the deadlift movement in the sagittal plane. The video-camera was mounted on a tripod (height about 1.20 m from the floor), positioned at a distance of about 1.60 m from the athlete (Figure 1B). To prevent participants from reconstructing the weight of the lifted load, and to avoid the size-weight illusion (36), the biggest disc (corresponding to 25 kg), which determines the dimensions of the load visible by the participants, was present in each condition. Furthermore, to prevent participants from seeing how many discs were charged on the barbell, the discs were covered by a black plastic cover. At last, the athlete's face was blurred to mask his expressions during the deadlift. Videos used in the experiment are offered in the online Supplementary Material.

#### 2.2.2. Experimental task

During the experimental task, participants observed the videos, sitting in front of the computer. Videos showing the lifting of each weight were displayed 16 times in a randomized order (3 weights x 16 times = 48 trials in total). Each video was followed by the question “Has he lifted the XX% of 1RM?”. Subjects were instructed to press as quickly possible the letter “v” for “Yes” answer and “n” for “No”. For each weight, in 8 of the 16 trials, the question asked to the participants mentioned the weight actually lifted by the athletes; thus, the correct answer was “Yes”. In the remaining 8 trials, the question mentioned the other two weights (4 times each), and thus the correct answer was “No”. Participants were not explicitly informed about how many different videos they will have to observe. The task duration was about 15 min. Participants took a pause after 24 trials (Figure 1C).

### 2.3. Data analysis

The age of participants was compared by means of Kruskal-Wallis tests since it was not normally-distributed. PL and CF's years of practice and 1RM value were statistically evaluated between groups by means of Mann-Whitney tests.

The main outcome parameter used to evaluate participants' responses was accuracy, which was expressed as the percentage ratio of correct responses (both when the right answer was “Yes” and “No”) to the total number of trials in 80%1RM, 90%1RM and 100%1RM. Furthermore, the coefficient of variation of the accuracy (CV) of each participant was also computed as the ratio between standard deviation and mean accuracy values obtained by averaging accuracy values in the three weight conditions.

Shapiro-Wilk test was applied to evaluate data distribution. Response accuracy was not-normally distributed, whilst the coefficient of variation was normally distributed. The within group analysis, aimed at evaluating the effect of the different percentage of weight (80%1RM, 90%1RM and 100%1RM) on response accuracy, was performed in each group by means of Friedman test, followed by *post-hoc* analysis. Kruskal-Wallis tests, followed by *post-hoc* analysis, was applied to compare the three groups (PL, CF and Controls) at each percentage of weight. For PL and CF, accuracy values were averaged across the three weight conditions and the resulting value were correlated with years of practice and 1RM values by means of Pearson correlation analyses. One-way ANOVA was applied on CV to evaluate the variability of the accuracy of participants in the three groups.

Normally distributed data are reported as mean values ± standard error (SE), while not-normally distributed data are given as median [interquartile range]. Statistical analyses were performed with SPSS Statistics 26 software. Significance level was set at 0.05. Effect sizes (*η*^2^ for normally-distributed data and Kendall's test—*W* and *r* value for not normally-distributed data) and 95% Confidence Interval (CI) were reported.

## 3. Results

The Friedman test showed a significant effect of weight in all groups. In PL (*χ*^2^(2, 18) = 9.41, *p* = 0.009, *W* = 0.26, CI [0.07, 1.00]) *post-hoc* tests showed that accuracy at 80%1RM (87.5 [72.9, 100] %) was significantly higher than that at 90%1RM (58.3 [39.6, 75.0] %, *p* = 0.016). In CF (*χ*^2^(2, 15) = 22.5, *p* < 0.0001, *W* = 0.75, CI [0.69, 1.00]) the accuracy at 90%1RM (41.7 [29.2, 58.3] %) was significantly lower than that at 80%1RM (83.3 [75, 83.3] %, *p* = 0.0007) and at 100%1RM (76.7 [75.0, 83.3] %, *p* < 0.0001). In Controls (*χ*^2^(2, 23) = 32.0, *p* < 0.0001, *W* = 0.70 CI [0.60, 1.00]) *post-hoc* analysis revealed that the accuracy at 90%1RM (41.7 [25–54.2] %) was significantly lower than that at 80%1RM (75 [58.3, 83.3] %, *p* = 0.0008) and at 100%1RM (75.0 [66.7, 83.3] %, *p* < 0.0001).

**Figure 2:**
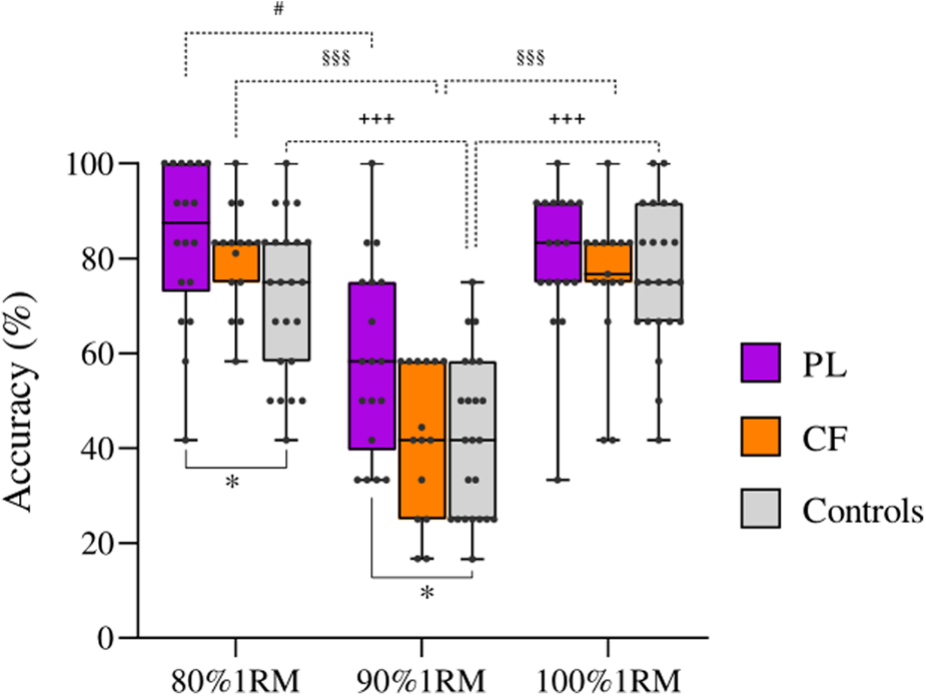
Response accuracy values at the three percentages of the 1RM displayed in the videos (80%1RM, 90%1RM, 100%1RM). Powerlifters (PL) are represented in purple, CrossFit® practitioners (CF) in orange and control participants in grey. The box depicts median and the 25th and 75th quartiles. The whiskers show the minimum and maximum. Points represent the accuracy of each participant. * Indicates a statistically significant difference between groups (*p* < 0.05). The significant within group differences among weights are indicated by # (within PL), § (within CF) and+(within Controls). # *p* < 0.05, §§§ and +++ *p* < 0.001.

The between groups analysis performed by means of Kruskal-Wallis tests at each weight revealed a statistically significant effect of group at 80%1RM (*H*(2, 56) = 6.22, *p* = 0.045, *η*^2^ = 0.08, CI [0.00–1.00]) and 90%1RM (*H*(2,56) = 7.68, *p* = 0.021, *η*^2^ = 0.11 CI [0.01–1.00]). In both conditions, *post-hoc* tests showed that the PL had a significantly higher accuracy than Controls (80%1RM *p* = 0.041; 90%1RM *p* = 0.021). No significant differences emerged between PL and CF (80%1RM *p* = 1.0; 90%1RM *p* = 0.09), and CF and Controls (80%1RM = 0.50; 90%1RM *p* = 1.0). No GROUP effect was found at 100%1RM (*p* = 0.52) (Figure 2).

No significant correlations were found between mean accuracy values (PL 74.2 ± 2.1%; CF 66.0 ± 2.6%) and 1RM in both PL (*R* = −0.11, *p* = 0.66) and CF (*R* = −0.24, *p* = 0.39), as well as between accuracy and years of practice (PL: *R* = −0.40, *p* = 0.07; CF: *R* = 0.29, *p* = 0.30).

The one-way ANOVA comparing CV values of the three groups (PL 0.29 ± 0.03, CF 0.35 ± 0.04; Controls 0.35 ± 0.03) did not reveal a significant group effect [*F*(2, 53) = 1.04, *p* = 0.36, *η*^2 ^= 0.19].

**Figure 3:**
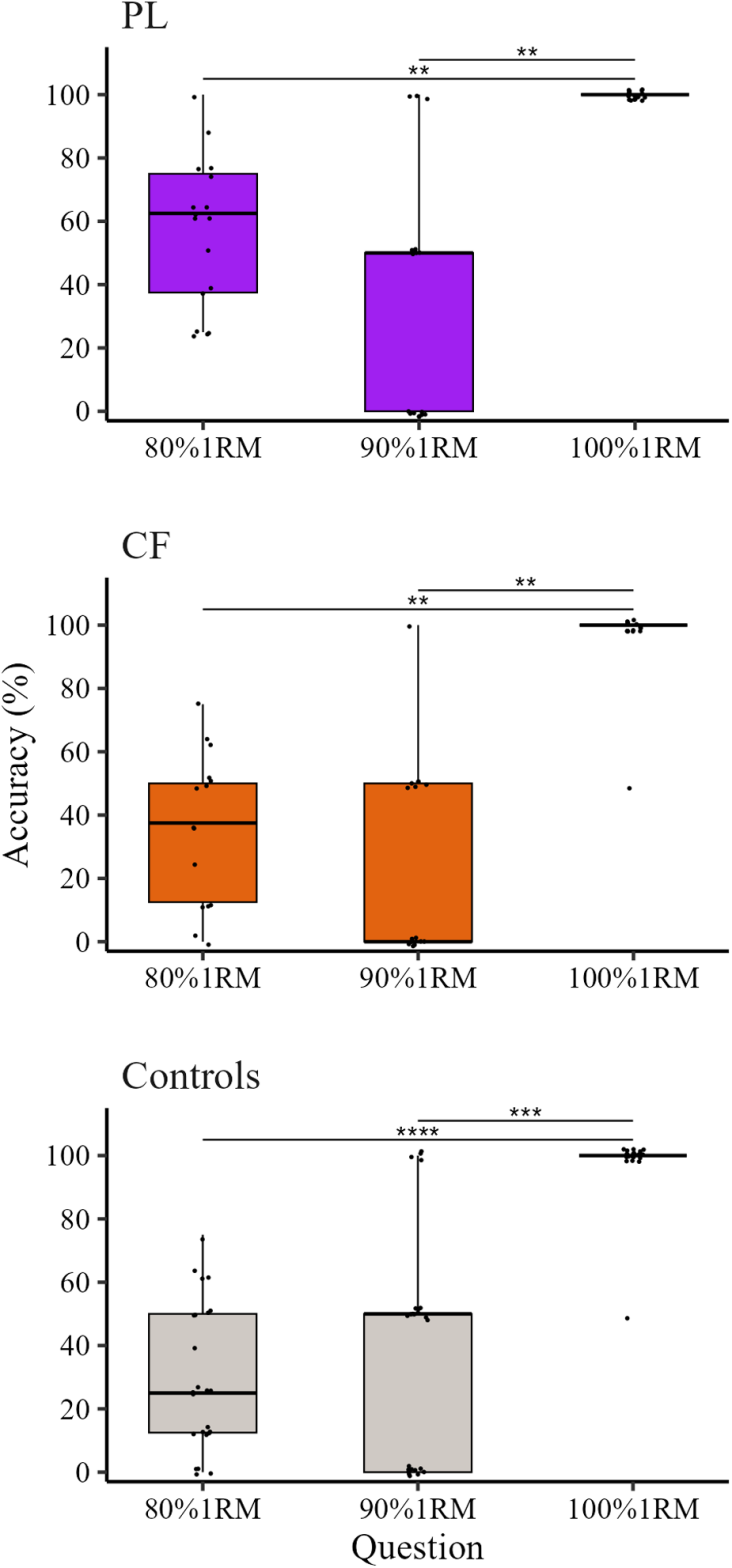
Response accuracy at 90%1RM when the question mentioned the three percentages of 1RM (80%1RM, 90%1RM, 100%1RM). Powerlifters (PL) are represented in purple, CrossFit® practitioners (CF) in orange and control participants in grey. The box depicts median and the 25th and 75th quartiles. The whiskers show the minimum and maximum. Points represent the accuracy of each participant. *** Indicates a statistically significant difference between groups (*p* < 0.001).

With the aim to specifically investigate if the weight of the barbell observed in 90%1RM was misattributed to either 80%1RM or 90%1RM or both, making the accuracy in this condition lower than in the other conditions, in each group Friedman test (followed by *post hoc*) was used to compare accuracy values at 90%1RM when the question mentioned 80%1RM (expected answer No), 90%1RM (expected answer Yes), and 100%1RM (expected answer No). In all groups, results showed a significant effect of QUESTION (PL *χ*^2^(2, 17) = 23.36, *p* = 0.0004, *W* = 0.69, CI [0.53, 0.90]; CF *χ*^2^(2, 15) = 20.70, *p* = 0.0007, *W* = 0.69 CI [0.42, 0.93]; Controls *χ*^2^(2, 24) = 28.66, *p* = 0.0002, *W* = 0.60 CI [0.44, 0.76]). *Post hoc* tests revealed that accuracy values when the question mentioned 100%1RM (100 [100, 100] % for all groups) were significantly higher than in case of 80%1RM (50 [0, 50] % for all groups; PL) and 90%1RM (PL 62.5 [31.25, 75] %; CF 37.50 [12.50, 50] %; Controls 25 [12.50, 50] %). No difference appeared between accuracy values when the question mentioned 80%1RM and 90%1RM. Data are represented in Figure 3.

## 4. Discussion

The aim of this study was to test the role of sensorimotor expertise in weight lifting in influencing the perceptual weight judgments during the observation of a sport-specific gesture. Results showed that only powerlifter were more accurate in evaluating the relative weight of the barbell with respect to non-experts. This difference was present when the weight lifted by the actor was below his 1RM, namely at 80%1RM and 90%1RM. No differences were found between PL and CF, and CF and Controls. Within groups, difference in the response accuracy were found among the different weights. No significant correlations were found between accuracy, 1RM and years of experience in deadlift. No differences among groups appeared in the coefficient of variation. When videos of 90%1RM condition were shown, the analysis on accuracy obtained as answer to the three questions showed similar values when the question mentioned 90%1RM and 80%1RM.

Results of the present study revealed that powerlifters, who had the highest and more specific expertise in deadlift, were more accurate in the perceptual weight judgments with respect to naïve participants. This was observed for weights below the 100%1RM, likely because the 100%1RM condition was markedly different from the other two conditions and the effort of the model appeared evident from his movement, causing a tremor during the lifting phase (see Videos in Supplementary Material). In this regards, Shim et al. (23) showed that visual information concerning the effort that a model exerts influences the observer's weight perception, and thus might have helped also naïve participants to infer the weight in 100%1RM condition. In studies using action observation paradigms, the role of motor repertoire was already shown to be crucial for other perceptual capacities such as the recognition of the actor identity (5, 6), the discrimination (3, 4) and the anticipation of movement (14). The present findings add a piece of knowledge concerning the mechanisms underlying the object relative weight perception, suggesting that motor resonance evoked in observer's played a crucial role. Indeed, when an individual observes an action in which she/he is an expert, the cortical motor system resonates with that action and a series of events, which influenced the following neurophysiological responses and behavioral performance, begin (17, 18, 37–39).

In the sport domain, one of the most famous studies was that of Aglioti and colleagues (19), which showed that basketball players predicted the success of free shots at a basket earlier and more accurately than coaches and journalists and had a time-specific motor activation during observation of erroneous basket throws. These results were interpreted as a consequence of athletes' ability to read the body kinematic features, characteristics that only the athletes' motor system was endowed with. In two more recent studies, it was shown that the sensorimotor skills acquired by means of years of practice in swimming (15) and soccer (16) helped athletes to predict the final outcome of the task and to infer the observed action's long-term intention, respectively. Therefore, the possibility for the observers' motor system to match the kinematics of the observed movement with the own sensorimotor representation was shown to be crucial in sport domain to anticipate both the fate of an action and the action's intention.

The innovative feature of the present findings is that the link between action and its sensorimotor representation was pivotal to evaluate a property of the object (i.e., the weight) involved in the observed action, confirming the initial hypothesis of the study. Previous studies, not involving athletes or people with peculiar abilities, proposed that the kinematics features of the movement are central to help the observers to infer it (21, 22, 27–29). This is in line with the principle of kinematic specification of dynamics postulated by Runeson and Frykholm, which states that the kinematic patterns of events contain information about the dynamic properties, including the weight of manipulated objects (20). Therefore, one might speculate that powerlifter, who developed a specific ability in deadlift, were better in judging the weight of the lifted load compared to naïve subjects thanks to their motor repertoire that includes the sensorimotor representation of this gesture. This highly detailed sensorimotor representation would allow PL to appreciate the subtle differences in performance model's kinematics that are at the basis of the recognition of the dynamic object's properties involved in the movement. It has to be noticed that this result could be motivated by the resonance evoked by the model, who was an experience powerlifter, in the observers who practiced Powerlifting. The results could have been different if the model in the video was a CrossFit® practitioner or a person naïve in deadlift. It is worth mentioning the role that the temporal features of movement might have played in helping participants to evaluate the relative weights of the barbell shown in the videos. As the weight of the barbell increased, the athlete's movement duration become higher. Thus one cannot rule out that video duration may have been a cue used by participants to determine that the lift was fastest, which is more likely with the lightest weight. Therefore, future studies on this matter might deeply explore the role of movement duration with respect to other kinematic features in estimating the weight of an object.

Furthermore, one cannot exclude that the motor resonance evoked in PL directly reflects force requirements of observed lifting actions, as suggested by Valchev and colleagues (40). At last, it cannot be ruled out that PL's perceptual experience influenced the results. Indeed, PL were used to perform, but also to observe other athletes performing the deadlift. This observational experience might have played a role in helping PL to evaluate the barbell relative weight. Whatever the case, the present results suggest that a specific sensorimotor expertise shapes motor resonance in such a way that the powerlifters gained the ability to judge the dynamic property of the objects involved in the observed movement.

The importance of having a specific ability in the observed gesture to be accurate in perceiving its features is further supported by the lack of difference between CF and Controls. In fact, while Powerlifting requires the athlete to perform a weight training over only three specific exercises such as the deadlift, the back squat and the bench press (30), CrossFit® includes within the same workout not only weight lifting training, but also metabolic and gymnastic exercises (31). Hence, although deadlift is a part of CF's training, in Powerlifting the higher training specificity and the largest amount of time spent on the deadlift might explain why only PL's accuracy was better than that of Controls.

Contrarily to the initial hypothesis, no differences appeared in the response accuracy between the two categories of experts, namely PL and CF. Indeed, despite in each weight condition PL's accuracy was numerically higher than that of CF, and a not significant trend (*p* = 0.09) appeared in 90%1RM, the differences were never significant. This lack cannot be attributed to the higher variability of one group with respect to the other, since the analysis of the coefficient of variation did not reveal any significant group effect. However, the variability of both groups was quite high as can be appreciated in Figure 2. This might explain the lack of difference between PL and CF. Future studies might also consider to specifically assess the difference among the technical features of deadlift when performed by PL and CF and correlate this aspect with perceptual weight judgment ability.

Furthermore, no significant correlation was found between accuracy and the level of expertise, here quantified by means of 1RM value (which was significantly higher in PL than CF) and years of practice. This finding is in contrast with previous results showing that having a motor or visual expertise explained the different corticomotor responses of basketball players with respect to coaches/journalists during the observation of a basketball free shots (19). The years and weekly hours of practice influenced the way a specific tool (i.e., tennis racket and epee) was integrated within the athlete's peripersonal space (41, 42) and regulated the hand blink reflex within the defensive peripersonal space in boxers (43). To explain these divergent results with respect the literature we cannot rule out that 1RM and years of experience may not be the most sensitive parameters to quantify the level of experience in this sport skills. This is a limitation of the present study and future works might consider other variables, maybe most related to the technical features of the deadlift.

When considering the effects of different percentages of the lifted weight, the 90%1RM was, for every groups, the hardest weight to distinguish. Indeed, in 90%1RM, PL's accuracy was significantly lower than that at 80%1RM, whilst the accuracy of CF and Controls was significantly lower than those at both 80%1RM and 100%1RM. An explanation could be that, since the 90%1RM is the condition in between the other two (10% difference with both 80%1RM and 100%1RM), participants might have been confounded and partly misattributed the weight of the 90%1RM to the other conditions. The results of the analysis on 90%1RM exploring accuracy values to the different percentage of 1RM mentioned by the question (80%1RM, 90%1RM, 100%1RM) suggested that participants often misattributed the 90%1RM to the 80%1RM. To corroborate this interpretation, it would have helped to ask participants not only to answer the question on the relative weight of the barbell, but, in case of negative answer, to request an estimate of the absolute weight. Unfortunately, this data was not collected and thus represent a limitation of the present study.

In conclusion, this study shows, for the first time, the role that sensorimotor expertise, gained during years of sport practice has in evaluating the property and, in particular, the relative weight of the objects involved in the observed movement. A fine sensorimotor representation of the sport gesture seems crucial to detect small changes in the observed movement kinematics that we speculate are at the basis of the recognition of the objects' property. To go deeper into the mechanisms regulating the role of the sensorimotor expertise in object weight estimation, future studies will need to decouple the effect of the observation of the mere kinematics information of the model (for instance, using point-light display technique) and that of the lifted objet (24, 25).

## Data Availability

The raw data supporting the conclusions of this article will be made available by the authors, without undue reservation.
